# Proton Pump Inhibitor Prescription in Nursing Home Residents: Prevalence, Appropriateness, and Associated Factors—A Secondary Data Analysis from Three German Regions and the Impact of Guideline Recommendations

**DOI:** 10.3390/ph17081082

**Published:** 2024-08-17

**Authors:** Ursula Wolf, Martina Wegener

**Affiliations:** 1 Pharmacotherapy Management, University Hospital Halle (Saale), 06120 Halle (Saale), Germany; 2 Institute of Health and Nursing Science, Medical Faculty, Martin Luther University Halle-Wittenberg, 06112 Halle (Saale), Germany

**Keywords:** proton pump inhibitor (PPI), prescribing behavior, nursing home residents, inappropriate prescribing, dosage, summary of product characteristics (SmPCs), guideline recommendations, drug approvals, patient safety, drug safety, secondary data analysis

## Abstract

Despite reliable evidence of adverse drug effects, the substantially increased prescription rates of proton pump inhibitors (PPIs) remain at a high level. This study analyzed the appropriateness of PPI prescriptions among residents of nursing homes in three regions of Germany. Baseline data of a cluster-randomized controlled trial were used to determine the prevalence of PPI prescriptions, the validity of indications, and the adequacy of the prescribed dosages according to 1. their drug approvals and 2. valid recommendation guidelines. Regression analyses were conducted to assess associated factors. A total of 437 residents in 37 nursing homes were included (mean age 83 ± 9.2 years, 72% women). The PPI prescription prevalence was 44% (n = 193). In 52/193 (27%) there was no adequate indication, and in 54 (39%) of 138 indicated PPI prescriptions it was overdosed. Yet, in only less than one-third (28%) of “adequate” prescriptions, the indication was according to the PPI approvals, whereas the majority (72%) were off-label indications in line with valid guideline recommendations. Non-indicated PPI prescription was associated with the total number of prescribed drugs (OR 1.32; 95% CI 1.18–1.62; *p* = 0.013). There were no associations with age, level of care dependency, cognitive impairment, prescription of psychotropic drugs, number of chronic diseases, number of physicians’ consultations, or study region. To conclude, in 55%, the high prescription prevalence among residents was either not indicated or overdosed. In total, only 20% (39/193) of cases of PPI use complied with the approved indications. There is a need for quality control of 1. PPI administration in German nursing homes, and 2. of guideline recommendations expanding the off-label PPI use by 72% within the indication scale, predominantly from wide prescription for low-dose ASA.

## 1. Introduction

Proton pump inhibitors (PPIs), introduced with omeprazole in 1989, are among the most commonly prescribed medications in the world [1]. In Germany, the frequency of PPI prescriptions increased steadily between 2007 and 2016, from 1.411 million defined daily doses (DDDs) to 3.8 million DDDs [2], remaining largely stable at this high level since then. However, this goes along with the 2009 and 2014 released German over-the-counter (OTC) PPI self-medication permissions and does not include the OTC PPIs, by which the overall consumption is assumed to continue to grow [3]. The elevated prescription rate of PPIs cannot be explained by an expansion in the number of corresponding diseases [2,3].

Although the valuable and highly effective PPIs are associated with considerable risk in long-term use, they remain referred to as generally well tolerated and are often used for prophylaxis and so-called “stomach protection”. Inappropriate use has been reported for up to two-thirds of cases [4]. PPIs are often prescribed without indication, in too high doses, and for too long. In particular, the non-indicated long-term use of PPIs without an indication must be viewed very critically. PPIs affect the gut microbiome by increasing the genera Enterococcus, Streptococcus, Staphylococcus, and the potentially pathogenic species Escherichia coli [5]. Regarding adverse drug reactions (ADRs), long-term use is associated with an increased risk of infections by, e.g., Clostridium difficile and Campylobacter [6,7], with evidence of the latter published as early as 1999 [6]. Deficiency symptoms of vitamin B12, iron, sodium, and magnesium increase with long-term use [8,9,10]. Associations between long-term PPI use and osteoporosis and fracture risk have also been described [11,12,13]. Long-term treatment was associated with an almost doubled increased risk of community-acquired pneumonia in a large cohort of older adults in primary care [14]. There are rare cases of agranulocytosis from PPI-induced hypersensitivity reactions, and reports suggest that PPI-induced neutropenia is immune-mediated, with evidence of cross-reactivity between PPIs [15]. The results of studies on the potential for cognitive decline with PPIs remain controversial [16,17,18] and further research is warranted. PPIs are also suspected of increasing the risks of chronic kidney disease [19] and myocardial infarction [20,21].

Against the background that, 1., despite being a vulnerable patient group, there are no special data on the German PPI prescribing behavior in the elderly nursing home residents, and, 2., more than 700 hundred self-conducted medication reviews within the EPCentCare study among nursing home residents show an alarming prevalence of PPI prescription, this study aimed to provide more insight into this prescription behavior in order to advocate more concrete restrictive or deprescribing practices perspectively.

The research focus was to determine the prevalence of PPI prescriptions in German nursing home residents, the appropriateness of the PPI indication according to both their Summaries of Product Characteristics (SmPC) approvals and to the national guideline recommendations and, furthermore, the adequacy of the PPI dosage for therapeutic and preventive purposes respectively according to their SmPCs [22,23,24,25,26,27,28,29,30,31,32,33,34,35,36,37,38,39,40,41]. Associations between inappropriate PPI prescription and resident and healthcare-related factors were analyzed. The presented data refer to a previous doctoral thesis [42]. In addition, the results of this selected study population were compared with the PPI prevalence in different own patient samples on PPIs. Finally, the current updated guideline recommendations were reviewed versus the previous national guidelines in terms of their potential forthcoming impact on future PPI prescription frequency [43,44,45,46,47,48,49,50,51,52,53,54,55].

## 2. Results

Out of 437 residents included in the analysis, *n* = 161 were located in Halle (Saale) (36.8%), *n* = 115 in Lübeck (26.4%) and *n* = 161 in Witten/Herdecke (36.8%), the eastern, western, and northern regions of Germany (Table 1).

Mean age was 83 ± 9.2 years, and 71.9% were women. The majority of residents were assessed as severe care dependent (level two out of three) according to an expert rater of the statutory health insurance system (Table 2).

**Figure 1:**
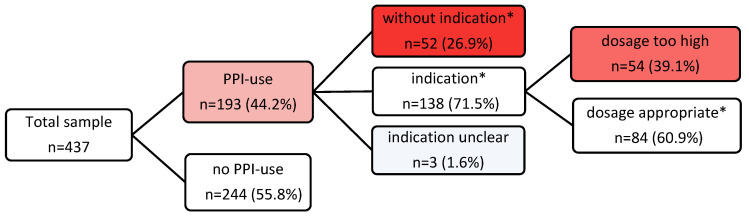
Flowchart of PPI prescription prevalence and appropriateness. * According to PPI SmPCs and/or guideline recommendations valid for the investigation period.

The prevalence of PPI prescriptions was 44.2% (*n* = 193), with pantoprazole being the most frequently prescribed (*n* = 153, 79.3%). The indication for PPI prescription was appropriate in 138 (71.5%) of the participants according to the valid SmPC drug approvals, which remained unchanged since the time of the study [22,23,24,25,26,27,28,29,30,31,32,33,34,35,36,37,38,39,40,41] (Table 3), and according to the documented diagnoses, entire medication lists, and guideline recommendations [43,44,45,46,47,48,49,50,51,52,53,54,55]. In 52 (26.9%) patients, the application was inadequate as it was without evidence of any indication. In three (1.6%) patients, the indication remained unclear. These three residents were on PPIs, although the nonsteroidal anti-inflammatory drug (NSAID) was an “on-demand“ medication. For temporarily prolonged NSAID use, the PPI indication would be adequate, but in the case of a single punctual application of the NSAID, the long-term use of PPIs would be inappropriate (Figure 1).

The differentiation was according to the list of diagnoses in each resident. The diagnoses list of a patient is available as a separate list and should characteristically contain all diagnoses and even historically relevant diseases. It exists independently of the medication list, but, for medications-related authorizations, you should always find the corresponding diseases and underlying indications for the prescribed drugs.

PPIs are generally prescribed as a statutory health insurance (SHI) prescription for the commonly insured patient. In principle, a drug can only be prescribed in Germany at the expense of SHI if it is used to treat diseases for which a marketing authorization has been obtained. However, there is a way to allow off-label use as a SHI benefit, so, doctors may be permitted to use medicinal products beyond the scope of the marketing authorization. There is also a possibility for an exceptional private prescription, and, in the case of PPIs, e.g., up to 20 mg pantoprazole by own OTC acquisition.

A detailed analysis of the prescriptions defined as “appropriate” showed that in less than one-third of the appropriate prescriptions, 39 (28.3%), the residents’ diagnoses had an approved drug indication, whereas the majority of prescriptions, 99 (71.7%), were prescribed according to the previous and current recommendations of the German guidelines [43,44,45,46,47,48,49,50,51,52,53,54,55] and in an off-label manner (Table 4). Almost 60% of patients aged ≥ 65 years (>60 in different versions and guidelines) received a PPI for low-dose ASA. As with the previous and currently updated PPI SmPCs, this was not an approved indication. In response to two independent inquiries about the approved indications for omeprazole and pantoprazole, the pharmaceutical companies confirmed that “ASA at low doses mainly inhibits platelet aggregation and is therefore used to prevent heart attacks and strokes. For this reason, ASA is classified in a separate group of drugs, the antiplatelet agents, and is not classified as an NSAID, although at high doses it has similar effects to NSAIDs. Long-term therapy with low-dose ASA generally does not require prophylaxis with proton pump inhibitors unless there are additional risk factors.” (1. MedInfo Germany. Authors’ Personal Medical-Scientific e-mail Correspondence. Pantoprazol AbZ 20 mg Magensaftresistente Tabletten. medical.affairs/teva.de/ratiopharm.de; 26 April 2024 and 2. MedInfo Germany. Authors’ Personal Medical-Scientific e-mail Correspondence. Omeprazol-ratiopharm NT 20/40 mg Magensaftresistente Hartkapseln. medical.affairs/teva.de/ratiopharm.de; 5 May 2024. Another company admitted to having no data on this topic (Medical information specialist. Authors’ personal medical-scientific e-mail correspondence. Pantoprazole NYC^®^ 20 mg, Takeda Pharma; 6 May 2024). Further off-label prescriptions resulted from guidelines recommending the combination of PPIs with any other anticoagulative drugs, predominantly involving direct oral anticoagulants (DOACs).

In addition, there are some recommendations from other national and international guidelines that were not relevant to the residents studied according to their documented diagnoses, e.g., no intensive care, trauma, and transplant conditions. Retrospectively, to compare the indication results, the “Appropriate Use of Proton Pump Inhibitors” by Phil Chung [56], and the current expert review [57], were applied to update with the latest, also international, guidelines.

**Table 4:** Indications for PPI prescription.

Aedequate Indications		Number = 138
Approved indication according to SmPCs *	Reflux esophagitis not defined further on	13 (9.4)
	Indicated by a past diagnosis in the history *******	12 (8.7)
	Gastric ulcer	4 (2.9)
	NSAID *** use + another risk factor	
	→ ibuprofen + age ≥ 65 years	3 (2.2)
	→ diclofenac + ASA ld ****+ age ≥ 65 (60) years *authors’ note: Cave*	3 (2.2)
	→ ibuprofen + ASA ld **** + age ≥ 65 (60) years *authors’ note: Cave*	1 (0.7)
	→ naproxen + prednisolone + age ≥ 65 (60) years	1 (0.7)
	→ ibuprofen + prednisolone + phenprocoumon + age ≥ 65 (60) years	1 (0.7)
	Eradication of Helicobacter pylori	1 (0.7)
	**Total number (%)**	**39 (28.3)**
Indications according to guideline recommendations ** **(entirely off-label use and meant for years or decades)**	*Use of antiplatelet drugs + another risk factor*	
	→ ASA ld ****+ age ≥ 65 years	81 (58.7)
	→ clopidogrel ****** + age ≥ 65 years *authors’ note: Cave* ******	3 (2.2)
	*Use of oral anticoagulants + another risk factor*	
	→ rivaroxaban + age ≥ 65 years	6 (4.3)
	→ apixaban + age ≥ 65 years	2 (1.4)
	→ phenprocoumon + age ≥ 65 years	2 (1.4)
	*Use of two platelet aggregation inhibitors*	
	→ clopidogrel ****** + ASA ld **** *authors’ note: Cave* ******	4 (2.9)
	*Use of an oral anticoagulant + antiplatelet*	
	→rivaroxaban high dose + ASA ld **** *authors’ note: Cave* *****	1 (0.7)
	**Total number (%)**	**99 (71.7)**

Values are absolute numbers (percentage). * Valid SmPC (Summary of Product Characteristics) approvals at time of investigation. ** Valid German guideline recommendations at time of investigation. *** NSAID non-steroidal anti-inflammatory drug. **** ASA 100 mg/day = ASA low-dose = ASA ld. Authors’ note: Cave: ASA ld in combination with NSAIDs no sufficient platelet aggregation inhibition. It is important to take ASA with a time delay before NSAIDs. ***** Authors’ note: Cave: in combination with DOACs clopidogrel should be preferred [58]. ****** Authors’ note: Cave: clopidogrel should not be combined with omeprazole due to insufficient prodrug activation. ******* A residual partial PPI indication from a previous condition with a prolonged PPI prescription.

Chung (Nebraska) [56] listed the spectrum of PPI treatment indication according to the drug approvals (Table 3) of the PPI SmPCs: PPIs are indicated for the treatment of the following conditions: “Zollinger-Ellison Syndrome, Barrett’s esophagus, acute upper GI bleed, erosive esophagitis, Helicobacter pylori treatment, gastric or duodenal ulcer, gastroesophageal reflux disease (GERD)”. 

For prophylactic PPI prescription, Chung summarized the spectrum as an overview we additionally compared retrospectively for the indication assessment results: “PPI’s are considered appropriate for the prophylaxis of UGIB in the following conditions: Mechanical ventilation for greater than 48 h; Coagulopathy defined as platelet count < 50,000/μL, INR > 1.5, or PTT 2x control; Traumatic head injuries with a Glasgow Coma Score ≤ 10 or inability to follow simple commands; Burns affecting > 35% of total body surface area; Major trauma with an Injury Severity Score ≥ 16; Spinal cord injury; Partial hepatectomy; Solid organ transplantation perioperatively in the ICU setting; Antiplatelet therapy (usually aspirin + clopidogrel, prasugrel, or ticagrelor) in patients at high risk for GI bleeding (prior history of GI bleeding; age > 60 years; concurrent use of anticoagulants, corticosteroids, or NSAID; Helicobacter pylori infection); Long-term NSAID use in patients with moderate to high risk of GI bleeding—Moderate risk is defined as 1 or 2 of the following risks: age > 65 years; high dose NSAID therapy (ibuprofen > 2400 mg daily, naproxen > 1000 mg daily, meloxicam > 7.5 mg daily); previous history of uncomplicated ulcer; concurrent use of aspirin, corticosteroids, or anticoagulants); High risk is defined as history of complicated ulcer especially recent, or >2 risk factors outlined in the moderate risk group; Any 2 of the following: Sepsis, ICU stay > 7 days, Occult bleeding lasting more than 6 days, High dose corticosteroids (>250 mg/day of hydrocortisone, >50 mg/day of methylprednisolone, >60 mg/day of prednisone, >10 mg/day of dexamethasone)”.

Overlapping with our procedure, he also points to the necessity of deprescribing upon discharge unless a chronic condition requires the PPI, to employ the lowest possible dose and shortest therapy duration [56], although concrete doses are not provided.

The data available for this secondary data analysis did not provide information going back to the start of the PPI prescription to distinguish whether it was a new prescription or a continuation after a hospitalization. It was only possible to determine the fact of PPI prescription and the corresponding diagnosis as an indication through corresponding approvals or national guideline recommendations.

Regression analysis for covariables on inappropriate PPI prescription revealed a significant association with the total number of prescribed drugs (odds ratio [OR] 1.32, 95% CI 1.18–1.62, *p* = 0.013) (Table 5). There were no other significant associations from the variables analyzed, neither for age, level of care dependency, number of chronic diseases, cognitive impairment, psychotropic drugs, number of physicians’ visits, nor the different regional study centers. However, there was some evidence of a higher rate of non-indicated PPI prescribing at the Witten/Herdecke study center (34%) compared to Halle (Saale) (22%) and Lübeck (20%).

The further review of all adequately indicated PPI prescriptions in terms of their correct dosage regimen, either a therapeutic or a prophylactic dose, showed that, in 54 participants (39% of indicated PPI prescriptions), the PPIs were overdosed respecting all documented diagnoses and comedications (Figure 1). This was typically the case for prophylactic PPI prescriptions as recommended in SmPCs and, above all, in the guidelines. It is precisely the guidelines, which make up the majority of these prescriptions, that are rarely accompanied by a specific dose recommendation in this prophylactic context, so that physicians may prescribe far too high doses for years or even decades based on the underlying condition of the recommendation. The overdose amounted to a two to fourfold PPI intake (Table 6). For these inadequately too high dose regimens, no statistically significant associations could be found with the covariables studied for the non-indicated prescriptions.

There was no tracking of the duration of PPI use, as this became influenced by medication review instructions. Because, as a consequence of these findings, the first author intervened and advised the respective physicians to adequately deprescribe the PPI in cases of non-indication and overdosage.

**Figure 2:**
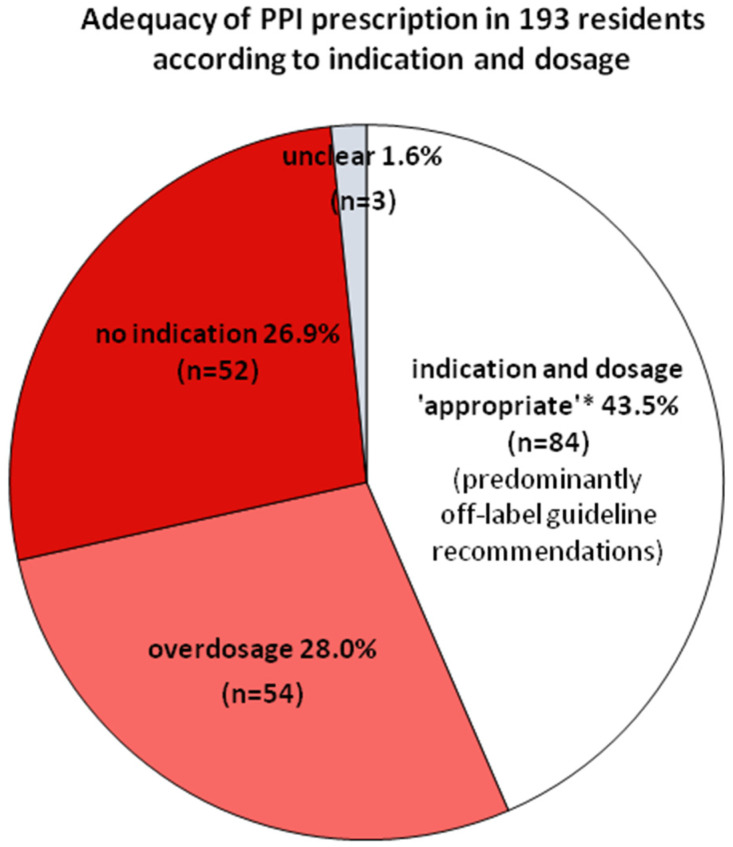
Prevalence of appropriate prescription*, non-indication, and overdosage among 193 residents on PPIs. In three cases, the indication remained “unclear” because NSAIDs were applied on demand without information on single doses or temporarily prolonged NSAID use. * According to the PPI SmPCs and/or guideline recommendations valid at investigation.

Figure 2 shows a summary of the differentially analyzed prevalences within PPI prescribing.

While these results refer to the selected study population of nursing home residents taking antipsychotics, we tried to place the results in the light of further and current prescription rates in different and more general populations. The first author was able to obtain a broad and ongoing real-world overview by reviewing daily ambulatory medication lists of hospitalized outpatients, now numbering more than 63,800, such as elderly patients ≥ 70 years of age undergoing trauma surgery after fractures. They provide further insight into the extraordinary PPI prescribing behavior:

1. Most recently, starting in January 2024, among 200 hospitalized trauma patients ≥ 70 years of age, primarily with hip fractures after falls, the ambulatory PPI prescription rate was 51% (102 patients) compared to 44% in the presented secondary data study. A slight improvement was seen in the prevalence of prophylactic 20 mg doses of pantoprazole (72% of all PPI prescriptions) versus the predominant 40 mg dose prevalence in the presented study. However, particularly in these trauma patients, the long-term PPI use-associated risk of osteoporosis and falls with fractures must be questioned as at least a partial adverse drug effect in this context.
2. An intervention study to improve polypharmacy in patients ≥70 years of age by Individual Pharmacotherapy Management (IPM) revealed a therapeutic 40 mg PPI prescription rate of 36% prior to the intervention, and an overall PPI prescription rate of 47%. The 40 mg dose was reduced to 24% with the IPM intervention [59]. To negate a confounding effect of nursing home residence or antipsychotic use on PPI overprescribing, as might be assumed from the secondary data of the presented study, it is important to note that, within the IPM-intervention study population of 404 patients, only 19% were nursing home residents, whereas 81% were home-dwelling elderly patients, and the mean antipsychotic prescription rate for the 404 study participants was 13%.

## 3. Discussion

In this analysis, almost half of the participants were prescribed PPIs. The study population comprised nursing home residents from the EPCentCare trial (subsequent recruitment and dropouts during the intervention contributed to the small differences in numbers in the final EPCentCare trial) [60]. This was a selective study cohort with at least one antipsychotic prescription and a consecutive medication review at initial recruitment. However, our results are consistent with previous international studies [61,62]. For example, Kelly et al. [61] found that 57.5% of 547 study participants had a PPI prescription, and Souto Barreto et al. [62], who studied 6275 nursing home residents, reported a PPI rate of 37.8%. The high rate of PPI prescription among nursing home residents was widespread in all three German regions studied, slightly more pronounced in the Witten/Herdecke study group. This high prescription rate is probably due to the apparent harmlessness of PPIs. As ‘stomach protectors’ they may also be assumed to prevent the patient from the adverse effects of other drugs.

About one-third of users received a PPI without an adequate indication. In residents with an indicated PPI regimen, only 28% of PPI prescriptions were based on a SmPC-approved indication. The vast majority (72%) of PPI indications were issued on the recommendations of the national guidelines and were therefore predominantly used on the basis and endorsement of these ‘in good conscience’ prescriptions although entirely in the off-label range. In particular, the guideline with the weak PPI ‘can’ recommendation [43] led to extremely frequent off-label prescribing of PPIs in residents ≥65 years of age on low-dose ASA, which accounted for almost 60% of all PPI ‘indications’. In the former German guideline version, there was a discrepancy between the text and the table for the important premise >1 risk factor and ≥1 risk factor to ‘can’ prescribe a PPI, which had far-reaching implications regarding age as a sufficient single risk factor with low-dose ASA. For the analysis of this study, the question of the inconsistent statements was addressed to the authors but remained unanswered. In the translated English version, the text and table content were unified to ≥1 risk factor hereafter. In addition, this guideline has been updated [44]: the 02/2016 version of the guideline recommended that “If a monotherapy with aspirin, another platelet aggregation inhibitor, NOAC, or VKA is given, PPI prophylaxis can be given if there is at least 1 risk factor for a gastroduodenal ulcer bleeding. Strength of consensus: strong consensus—recommendation”. At that time, age ≥ 65 years counted as at least one risk factor. The recommendation modified in the updated 2021 version stated “If monotherapy with ASA, a P 2 Y 12 inhibitor, DOAC or VKA is administered, PPI prophylaxis should be given if at least one other risk factor for the occurrence of a gastroduodenal ulcer and/or ulcer complication (see ulcer complication (see Statements 7.3 and 7.4) is present. If only the risk factor age > 60 years and no other risk factor is present, prophylaxis is not necessary” [44]. This currently valid recommendation sounds weaker, but the age has been lowered even further and the “can” has been changed to a “should” recommendation in case of any second risk factor, e.g., diabetes mellitus. What makes the scenario even more confronting is the fact that this indication typically means PPI for years and decades of life in low-dose ASA. Additionally, frequently, an adequate indication for the ASA prescription itself was not obvious in our patient population. Low-dose ASA for the primary prevention of cardiovascular disease is still being studied in terms of risk/benefit, and trial data remain controversial; the decision should be made on a more precise individual-patient basis [63]. Other studies have also shown that PPIs are often prescribed as prophylaxis due to the use of low-dose ASA. A French study in hospitalized elderly patients revealed that about 60% of PPI prescriptions were not in accordance with the French guidelines; the leading mismatch was primary prevention for low-dose ASA [64]. A second leading noncompliant indication was lengthy of treatment without reevaluation, the mean duration of PPI prescription was 2.3 years and exceeded 6 months in 62% of cases [64]. Although this German updated guideline on the prophylactic use of PPIs with low-dose ASA no longer recognizes age alone as a risk factor, but only in combination with other factors, such as underlying severe diseases, the recommendation been strengthened from “can” to “should” and the age limit lowered from 65 to 60 years. This means PPIs until the end of life in these patients on low-dose ASA, despite all the known ADRs manifesting as a result of long-term use, such as osteoporosis with increased hip fractures [11]. According to the results of this UK study, the incidence of associated hip fractures increased steadily with a longer duration of PPI prescription [11]. A recent guideline of the German Society of Internal Medicine (DIM) recommends that “in monotherapy with an antiplatelet agent (low-dose ASA or another platelet aggregation inhibitor), a PPI should not be prescribed as a rule. Strength of consensus: strong consensus. Justification: The increase in the risk of bleeding due to low-dose long-term low-dose ASA therapy is low. PPI administration is therefore not indicated in all patients, but only in patients who have at least one risk factor for gastroduodenal ulcer bleeding. Risk factors include, for example, age ≥ 65 years, a history of ulcers, concomitant bleeding-inducing medication, a severe course of a general illness (e.g., type 2 diabetes), smoking, or an H. pylori infection” [52]. Again, we have the off-label indication in patients ≥ 65 years of age taking low-dose ASA as the only risk covariate, which in turn includes the vastly expanded patient population for whom this recommendation foresees years and decades of PPI use. The recommendations in these guidelines need to be adjusted to a patient-centered, balanced level with individualized, ongoing benefit–risk assessment and reassessments. Regarding the duration of PPI prescription, this aspect was not considered in the present study, although this is an additional serious factor of inappropriateness [11]. Further complicating the liberal PPI over-prescribing, Haasturp et al. ascertained that the definition of long-term PPI use was rationalized in only 20% of the studies evaluated and ranged from >2 weeks to >7 years [65], with most >1 year and >6 months; although the approved indication for a prolonged therapy is only in very rare diseases such as with Zollinger–Ellison syndrome [22,23,24,25,26,27,28,29,30,31,32,33,34,35,36,37,38,39,40,41].

Regression analysis revealed that the increased number of drugs administered daily was significantly associated with non-indicated PPI therapy. The association between non-indicated PPI prescriptions and polypharmacy has been reported by various other studies [62,66], the high PPI use almost representing a prescribing cascade [66].

In addition to the discussed guideline-indicated off-label PPI prescriptions, the great amount of PPI use without any indication (27%) should be regarded as very critical. Although PPIs are generally considered well-tolerated drugs, significant ADRs might occur, especially in long-term therapy. The present study could not investigate whether the prescription of non-indicated PPI was a result of previous hospitalization, but this can partly be assumed as various studies have demonstrated an increase in prescriptions of PPIs after hospitalization. For example, the analysis by Scheurlen et al. revealed that failure to discontinue PPI after hospitalization is one of the major factors in non-indicated long-term PPI therapy [67]. In accordance, also Ahrens et al. reported, that non-indicated PPIs continue to be prescribed by family physicians after hospital discharge [68]. In order to prevent these non-indicated prescriptions, it has been recommended by a general practitioner guideline on multi-medication to provide information on the duration of the medication listed in the discharge letter in the context of discharge management [47].

Not least to complete the PPI overuse spectrum, within the indicated PPI prescriptions, 39% of the administration regimen was overdosed contrary to the PPI approvals. The reason for overdosing of PPIs might be the assumption that the higher the dose the greater the gastrointestinal protection, although there are only a few approved strong therapeutic indications for higher doses [22,23,24,25,26,27,28,29,30,31,32,33,34,35,36,37,38,39,40,41], as can be seen from the overview in Table 3. Furthermore, the guideline recommendations predominantly do not specify any dosage. The prevalence of overdosage is consistent with the results of other studies, e.g., a report indicating that 41.6% of study participants were prescribed an overdosed PPI [61]. In a retrospective cross-sectional study including adult patients of 1006 general and 39 gastroenterological practices in Germany, Plehhova et al. also emphasized the discrepancies between mild indications and high-dose or long-duration PPI [69]. Dosage recommendations should not exceed the dosing regimens according to the drug approvals [45,52].

Of the currently available prescription PPIs, pantoprazole, omeprazole, esomeprazole, lansoprazole, dexlansoprazole, and rabeprazole, the SmPc for dexlansoprazole indicates the potential need for dose adjustment and adherence to an upper dose limit due to the reduced elimination of lansoprazole in elderly patients [22,23]. The reduced elimination of lansoprazole in elderly patients relates to the elimination half-life being prolonged by approximately 50 to 100% [25]. The absence of an increase in maximum plasma concentrations does not eliminate the problem of a higher exposure. Similarly, the area under the curve (AUC) after seven days of 20 mg rabeprazole sodium daily was almost double that of young healthy volunteers [40,41]. Does it really outweigh the increased risks of ADRs stating that “However there was no evidence of rabeprazole accumulation” [40,41]? This is more an exclusion of an intoxication risk. Even the SmPCs of PPIs, such as for esomeprazole, e.g., that do not indicate dose adaption in the elderly, warn that the “benefits of use of PPIs should be weighed against the increased risk of fractures as patients in this category may already be at high risk for osteoporosis-related fractures. If the use of PPIs is required, they should be managed carefully according to established treatment guidelines.” [27]. As one of the pathophysiological mechanisms of PPI-induced osteoporosis is reduced vitamin D uptake, appropriate substitution should be provided on an individual basis.

In the context of overdose, there remains a numerically less prevalent and, in prescription routine, unconsidered aspect. Approximately 3% of the Caucasian population, but 15–20% of the Asian population, do not have a functional cytochrome p450 2c19 (CYP2C19) enzyme, and are therefore poor or slow metabolizers. The CYP2C19 isoenzyme, which is involved in the metabolism and degradation of all available PPIs, exhibits genetic polymorphism, which is less pronounced for pantoprazole or esomeprazole than for omeprazole. After repeated once-daily oral administration of 20 mg omeprazole, the average AUC in poor metabolizers was from approximately 5 to 10 times higher than in individuals with a functional CYP2C19 enzyme. Average peak plasma concentrations were also from three to five times higher. However, these results have no implications for the dosing of omeprazole [29]. A study of the prevalence of PPI ADRs in these patients, especially in long-term users compared with regular PPI metabolizers, would be of interest. In affected individuals, the omeprazole metabolism is assumed to be catalyzed by CYP3A4, the second enzyme involved in the omeprazole degradation process. After a single dose of 40 mg of pantoprazole, the mean AUC was approximately six times higher in poor metabolizers than in persons with intact CYP2C19 enzyme activity, and the mean maximum plasma concentration was increased by about 60%. Strikingly, these results also remain classified as irrelevant to dosing in their SmPc [35] despite the increasing global attention given to the risks of PPI overdose. As early as 1996, Kuipers et al., analyzing ADRs of long-term high-dose omeprazole in patients with reflux esophagitis and H. pylori infection, also indicated an increased risk of atrophic gastritis [70], which has been regarded as a precursor of gastric cancer. Another study has demonstrated that, even after H. pylori eradication, long-term PPI use remained associated with more than a twofold increased risk of gastric cancer [71]. Presumably, this may be particularly relevant in the Asian population with a higher burden of gastric cancer. RCTs to better establish a causal relationship between long-term PPI use and gastric cancer are required. An enduring substantial PPI-induced suppression of gastric acidity with subsequent increased hypergastrinemia, bacterial overgrowth, and gastric atrophy might be pathophysiologic processes to be accounted for.

Early this month, Plehhova et al. published the concerning German data on the growing market of PPIs, indicating a substantial amount is based on prescriptions. The ongoing increase in OTC PPI purchases, as already assumed in the introduction of the present study, was confirmed, further evidencing a recent increase in prescriptions. Notably, PPIs were predominantly sold in the largest package sizes of more than 90 tablets and remarkably often in their highest strength of 40 mg, even for omeprazole, which is twice the DDD [72]. In their preceding study on PPI overuse, they reported an average PPI treatment duration of 141 days, and 59% of PPI prescriptions were in patients > 60 years of age [69]. PPIs are among the most overused drugs in the world, for which limited knowledge of ADRs, polypharmacy, poor regulation, and financial influences have been identified as the main reasons [73].

Completing the three study group regions with the results presented, analogously a study in Bavaria, in southern Germany, confirmed that an appropriate on-label indication was lacking in 52.0% of initiating PPI users [74]. Liu et al. also found that, in 47% of cases, unapproved indications accounted for new PPI users [75]. Strikingly, in this Asian study group, the prevalent PPI was omeprazole, despite the fact that the Asian population is obviously even more severely impacted by the high prevalence of CYP2C19 polymorphism compared to pantoprazole and probably needs lower doses. As discussed above, this is particularly noteworthy in the context of the overall higher incidence of gastric cancer already preexisting in these populations.

The results of this study highlight that the prescription of PPIs is handled far too carelessly in multiple respects. Overlapping results have been complained about in various other countries, and very similarly in Spain by Savarino et al., who stated that “The major reasons for the misuse of PPIs are the prevention of gastro-duodenal ulcers in patients without risk factors and the stress ulcer prophylaxis in non-intensive care units, steroid therapy alone, anti-platelet or anti-coagulant treatment in patients without risk of gastric injury and the overtreatment of functional dyspepsia” [76]. Analogously, for inappropriate use even over more than 8 weeks in about 40% of outpatients in an Italian study the highest rates were observed for the treatment of dyspepsia and anti-coagulant therapy, most frequently although less inappropriately for gastroesophageal reflux disease and prophylaxis of anti-platelet/nonsteroidal anti-inflammatory drugs [77]. It is beyond any rationality that the alarming and dubious rates of PPI prescription by physicians continue to rise, having already manifested in mass consumption of these drugs worldwide, ignoring all the cautionary study results and even the PPI advices obligatorily specified by the SMPCs, which states that “Patients should be prescribed PPIs at the lowest dose and for the shortest duration required for the condition being treated and be reassessed to ascertain whether continued PPI therapy remains beneficial” [23]. “Patients should be warned about additional risks with long-term use of the medicinal products and the need for prescription and regular surveillance should be emphasized” [39]. “In geriatric patients > 71 years of age benefits of use of PPIs should be weighed against the increased risk of fractures as patients in this category may already be at high risk for osteoporosis-related fractures. If the use of PPIs is required, they should be managed carefully according to established treatment guidelines” [27]. But, furthermore, the applicability of the established treatment guidelines has to be assessed in each individual patient condition as well, especially in the elderly. For example, according to a review, predominantly affected geriatric patients with chronic diseases are also at increased risk for symptoms of loss of muscle function due to PPI-induced magnesium deficiency [78], which, in addition to PPI-induced osteoporosis, may enhance the risk of falls and fractures, particularly in the susceptible elderly PPI-prescribed patients.

Against the background of the study results strengthened by a broad literature update, there is an unsolved urgent need for an intervention aimed at optimizing PPI prescription. Any inadequate prescription should be avoided to ensure the safety of drug therapy. According to the PPI-deprescribing guidelines for risks of a rebound phenomenon, the step-down procedure in deprescribing PPIs involves a fortnightly 50% dose-reduction [79], with consequent observation of the patient and the symptoms. Gradual dose reduction via transitioning from a double to a single dose, halving the single dose, and alternating the dose every second day is usually applied. The optimal time between steps has not been studied. Using PPI on-demand may be an equally strong option [79]. In patients with reflux symptoms or chest pain reflux symptoms who do not respond satisfactorily to PPI therapy, at least a pH-metrically controlled PPI therapy is recommended [80]. And to confirm or rule out gastroesophageal reflux disease (GERD), in patients with PPI-resistant symptoms, ambulatory pH impedance monitoring should preferably be performed after discontinuation of PPI therapy [81]. According to the Lyon Consensus, the conclusive diagnosis of GERD and therapeutic strategies should be based on the analysis of the patient phenotype based on further investigations [82]. In a double-blind study of 100 patients, PPI therapy was discontinued in 34 patients, supporting the early reflux monitoring of acid suppression to phenotype the patient with inadequate PPI effect and thus provide personalized care and avoid unnecessary PPI overuse [83]. The strongest predictor was the absence of pathological acid exposure as measured by wireless pH measurement after at least 7 days of PPI abstinence. The results of a retrospective cross-sectional German primary care databased analyses on an extensive sample size of 472 146 patients indicated that the majority, 92.2%, could benefit from PPI dose reduction, 62% did not need PPIs any longer, 44% received inappropriately prescribed PPIs, and 42% would benefit from an alginate add-on [69].

In several countries, such as the United States [84], France [85], and Italy [86], there have been studies and efforts to publish position papers on the problem of PPI overuse. A recent Canadian investigation also found that only one-third of PPI prescriptions in ambulatory geriatric patients were appropriately indicated. Given their susceptibility to ADRs, these patients should be a priority target group for PPI deprescribing initiatives [87]. A recent systematic review of global trends and practices in PPI use, focusing on 65 articles with 28 million PPI users in 23 countries, found that about 1/4 of adults use a PPI, 63% of whom were <65 years of age. Of further concern was the finding that almost 2/3 of PPI users were on high doses, 25% of users remained on PPIs for >1 year, and 28% of these for >3 years. The New Zealand study group of Shanika et al. concluded that these alarming findings should serve as a “catalyst” for more rational prescribing, especially for long-term use [88].

Since there is no doubt that PPIs, e.g., are effective in preventing upper gastrointestinal ulcers and bleeding associated with low-dose ASA in patients at risk [89,90], as is, e.g., obvious in more vulnerable patients aged ≥75 years [89], the corresponding precise challenging responsibility of physicians and also patients themselves is to identify individual patient-centered risk symptoms so that PPIs are not dispensed to the entire population who are on low-dose ASA. The frequency of high-dose or long-term PPI prescriptions to patients with mild indications or merely for prophylaxis deserves special attention, e.g., also in gastroesophageal reflux disease. And, for example, when treating pure reflux symptoms, the guideline should also apply to the PPI approval, stating that, e.g., pantoprazole 20 mg “is indicated for short-term treatment of reflux symptoms (e.g., heartburn, acid regurgitation) in adults. The recommended dose is 20 mg of pantoprazole (one tablet) per day. It might be necessary to take the tablets for 2–3 consecutive days to achieve improvement of symptoms. Once complete relief of symptoms has occurred, treatment should be discontinued. The treatment should not exceed 4 weeks without consulting a doctor. If no symptom relief is obtained within 2 weeks of continuous treatment, the patient should be instructed to consult a doctor.” [39]. For cardiological indications supported by ongoing studies on the most effective and least harmful anticoagulation in patients with atrial fibrillation (AF) and various types of chronic ischemic heart disease, such as chronic coronary syndromes after stenting, DOACs are preferred as long-term antithrombotic therapy, plus clopidogrel, while ASA is no longer used on a long-term basis [58]. The OLTAT registry searching for optimal long-term antithrombotic treatment of patients with stable coronary artery disease and atrial fibrillation revealed that, after 5 years, the add-on of antiplatelet therapy to oral anticoagulants was independently associated with a higher risk of bleeding and overall mortality, without significant reduction in cardiac and cerebral ischemic events [91]. Following the current perspectives, the triple therapy regimen with a DOAC in chronic anticoagulation should only include low-dose ASA for up to 7 days or until hospital discharge, and a P2Y12 inhibitor for 6 to 12 months, depending on the risk of thrombosis after percutaneous coronary intervention with drug-eluting stent placement [92]. According to a recent systematic review and meta-analysis in patients with atrial fibrillation and stable ischemic heart disease, DOAC plus single antiplatelet therapy (SAPT) is associated with a significant increase in hemorrhage without a significant reduction in thrombotic events, cardiovascular mortality, or all-cause mortality compared to DOAC monotherapy [93]. Thus, there has been an apparent overuse of antiplatelet agents, needing reduction according to the results of these studies. This, in consequence, would mean less risk of bleeding by reducing the use of potential hemorrhage inducers, such as low-dose ASA, and represents another important lever to reduce prophylactic PPI prescribing.

The WHO Global Patient Safety Action Plan 2021–2030 to eliminate preventable harm in health care is another challenge in the ongoing efforts, including the improvements in patient and drug safety, that have been underway for decades [94]. Every healthcare professional and, ideally, empowered patients, should take personal responsibility with regard to the massive PPI overuse.

### Strengths and Weaknesses

The present analysis focused not only on prescription prevalence but also on the indication and dosage of PPIs among nursing home residents in order to provide a more comprehensive and precise insight into their use of PPIs. A further particular strength of the study is that indications have been differentiated between German PPI drug approvals and information and indications based on earlier and current German guideline recommendations that partly include weak recommendations leading to off-label use. The individual indication, as well as the appropriateness of individual dose levels, were examined independently by two researchers, a pharmacist and a specialist in internal medicine, both with pharmacological expertise (authors M.W. and U.W). Their independent reports on indications and dosages were 100% consistent.

By analyzing study participants from 37 nursing homes in three different regions of Germany, the northern, eastern, and western parts, the prescribing behavior of physicians from different regions leads to some generalizability for German prescribing routines in elderly patients.

As a limitation, due to secondary data analysis, only the residents with at least one antipsychotic prescription were included. However, the assumption of a confounding effect may be contradicted by the authors’ own data from other independent analyses of PPI prevalence, which were provided additionally.

The data collection followed a standardized protocol. Data were extracted from the residents’ records and therefore inconsistencies in routine data documentation and patient status cannot be ruled out.

For the purpose of this cross-sectional secondary data analysis, only baseline data were evaluated. The duration of the PPI prescription and possible changes in the medication prescription regimen during the course of the study were not further taken into account.

Despite individual risks due to impaired hepatic function or comedication and polypharmacy are a significant composite and were addressed in the medication reviews of the nursing home participants, we do not refer to the even major pharmacokinetic and pharmacodynamic PPI-drug interactions, such as for omeprazole and clopidogrel [95,96,97,98,99], or the increase in citalopram with omeprazole, in this data analysis. In addition, PPIs may reduce the absorption of active substances whose bioavailability is pH-dependent, a risk not referred to in the study data presented.

Although the results of this study, based on a relatively small number of participants, provide deeper insights and are supported by other global findings on PPI overprescribing, larger numbers might strengthen the evidence.

## 4. Material and Methods

The reported study is a secondary data analysis of the EPCentCare study [60,100], which included a total of 1042 residents of 37 nursing homes at baseline. The PPI data refer to the participants of the EPCentCare study group in terms of both conditions, residents with an antipsychotic prescription and those who received a medication review, based on the medication data in the residents’ records (including hospitalization and discharge letters, physicians’ visitation documentation). Baseline data from this subsample (*n* = 437) were included in this secondary data analysis. Of these, the following variables were examined:

- At the individual level: age, level of care dependency, diagnoses including kind and number of chronic diseases, cognitive impairment, number of physician contacts (general practitioner and/or specialists), and number of prescribed drugs in the long-term medication; drug groups: antidepressants, neuroleptics, anti-dementia drugs, and antiparkinsonian drugs
- At the cluster level: the different study centers for regional differences.

The data were collected between November 2014 and October 2015. The detailed review of each PPI indication or missing indication according to the recorded diagnoses and medication list was independently conducted by an internist and a pharmacist. The results were compared with the PPI prevalence in different own patient samples and with the updated guidelines for their potential future impact on PPI prescription.

Statistics were performed using STATA software, version 13. Categorical variables were described by absolute and relative frequencies. For continuous variables, the Shapiro–Wilk test was applied to determine whether they had a normal distribution so that the mean and standard deviation or the median and two quartiles could be reported. Binary logistic regression was applied to analyze the association between variables.

## 5. Conclusions and Way Forward

In this study, the high prevalence of 44% of PPI prescriptions was either without an appropriate indication or overdosed in 55% of nursing home residents. Within the ‘appropriate indications’ an approved indication reflected the smallest proportion of PPI prescriptions. The predominant spectrum of indications was extremely expanded by a number of weak guideline recommendations that must be questioned on a patient-by-patient basis. These expert recommendations, although evidence-based, strongly contribute to the widespread prescription of PPIs for off-label uses, as demonstrated for the low-dose ASA-based indication in the elderly. The corresponding updated guideline recommendation is a bit less liberal, but parallel guidelines came up from other medical societies that return to the questionable weak indication. There is an urgent need for an intervention to promote the appropriate prescription of PPIs.

1. A patient-centered structured medication review including all relevant patient and medication scores is required at regular intervals for individual pharmacotherapy management [59] in order to avoid both individual non-indication and overdosing. As a contribution to patient and drug safety, this could prevent unnecessary initiation, overdosage, and long-term use of PPIs, with the risks of associated ADRs. 2. The various guideline recommendations should be homogenized and provide a clearer strategic orientation, whereby the applicability to the actual medical condition of the individual patient and the changes in long-term follow-up care obligates regular reassessments. 3. Deprescribing requires stringent adherence to the regulations to avoid rebound [79], and the use of on-demand PPI or intermittent PPI [101], as well as transient alginate, may aid in this process [102]. 4. Not least for the concomitant use of OTC PPIs, patient training to strengthen co-responsibility, and patient empowerment with regard to ADRs, could be an effective measure to change the partially nonchalant attitude in this regard. Depending on the individual situation, especially patients with symptomatic gastroesophageal reflux disease and nocturnal reflux symptoms should also be advised to pursue non-pharmacologic accompanying measures, such as avoiding chocolate, caffeine, spicy foods, citrus fruits, and carbonated beverages, pursue smoking cessation and alcohol reduction, avoiding late meals and raising the head, avoiding abdominal breathing, and pursuing weight loss in the case of obesity [45,46]. 5. The prescribing practices of attending physicians are the domain in this critical issue, and education and audit feedback initiatives may help, as may clear deprescribing instructions in hospital discharge letters and adequate diagnostic procedures in clinically dubious cases [81]. 6. Health insurance companies should question off-label prescriptions as they do in other more expensive medical therapies. 7. The fact that a widely consumed drug is released for OTC availability, despite being mostly used for off-label conditions, needs to be re-evaluated. Saving the prescribing physician’s budget, as may result from regulations in the German health care system, also via private prescriptions, should not be the reason. 8. The national and international drug regulatory and supervisory authorities BfArM/EMA/FDA must subsequently promote the concrete distinction between low-dose ASA as an antiplatelet agent and standard-dose NSAIDs in the PPI-SmPCs, because, considering the far-reaching consequences, lifelong PPI intake for low-dose ASA is not routinely necessary in all these patients, and each individual indication requires careful and repeated evaluation.

## Figures and Tables

**Table 1:** Clusters and participants per study center.

Study Center	Nursing Homes (Clusters), *n*	Participants per Study Center, *n*
Halle (Saale)	12	161
Lübeck	12	115
Witten/Herdecke	13	161
**Total**	37	437

**Table 2:** Baseline characteristics *n* (%) of study participants (*n* = 437).

**Age**, years, mean (SD) [range]	83 (9.2) [77–89]
**Female**	314 (71.9)
**Marital status**	
Widowed	231 (52.9)
Married	118 (27.0)
Unmarried	44 (10.1)
Devorced or separated	44 (10.1)
**Length of residence (weeks)** median (2 missings)	115.6
Level of care dependency *	
None	2 (0.5)
0	5 (1.1)
1 (considerable)	92 (21.1)
2 (severe)	209 (47.8)
3 (most severe)	129 (29.5)
Nutritional status, BMI ** (1 missing)	
Severe malnutrition	6 (1.4)
Malnutrition	11 (2.5)
Normal	196 (44.9)
Pre-obese	143 (32.7)
Obese	80 (18.3)
Cognitive impairment (DSS > 4) ***	283 (64.8)

* Level of care dependency categories: residents’ need for care was assessed by the medical service of the German social care insurance; need for care in performing activities of daily living and household tasks was defined as Level 0: <90 min/day, Level 1: at least 90 min/day, Level 2: at least 3 h/day, and Level 3: at least 4 h/day. ** Intended as an initial indicator only, estimated using the WHO BMI categorization without anthropometric, biochemical, clinical, and dietary assessments. *** DSS: Dementia Screening Scale (total score ranges from 0 to 14; higher scores indicate more severe cognitive impairment).

**Table 3:** Example overview of the two prescribed PPIs omeprazole and pantoprazole for approved indications and dosing per day (/d) in adults as SmPCs documented at the time of the study and unchanged today (except for the treatment dosage of NSAID-associated gastric and duodenal ulcers).

Approved Indications	Omeprazole *	Pantoprazole **
Treatment of duodenal ulcers	20–40 mg/d, 2–4 weeks	40–80 mg/d, 2–4 weeks
Prevention of relapse of duodenal ulcers	10–20 mg/d (40 md/d)	20–40 mg/d
Treatment of gastric ulcers	20–40 mg/d, 4–8 weeks	40 (80) mg/d, 4–8 weeks
Prevention of relapse of gastric ulcers	20 mg/d (40 mg/d)	20–40 mg/d
In combination with appropriate antibiotics, Helicobacter pylori eradication in peptic ulcer disease (consider national recommendations)	2 × 20 mg/d or 1 × 40 mg/d, 1 week	2 × 40 mg/d (second tablet 1 h before evening meal), 1 (2) weeks
Treatment of NSAID-associated gastric and duodenal ulcers	20 mg/d, 4 weeks (8 weeks)	20 mg/d (updated 40–80 mg)
Prevention of NSAID-associated gastric and duodenal ulcers in patients at risk ***	20 mg/d	20 mg/d
Treatment of reflux oesophagitis	20–40 mg/d, 4 weeks (8 weeks)	40–(80) mg/d, 4 weeks (8)
Long-term management of patients with healed reflux oesophagitis	10–40 mg/d	20 mg/d
Treatment of symptomatic gastroesophageal reflux disease (GERD)	10–20 mg/d, 4 weeks	20 mg/d 2–4 weeks (8), try on demand regimen hereafter
Treatment of Zollinger–Ellison syndrome and other pathological hypersecretory conditions	Individually adjusted dose 60–80 mg/d, for 120 mg devide 2 × 60 mg/d; maintenance dose 20 mg/d or higher	80 mg/d initial dose, titrate hereafter; doses >80 mg divide and give twice daily; transient >160 mg/d possible

* SmPC omeprazole [29,30,31,32,33,34], ** SmPC pantoprazole [35,36,37,38,39]. *** Prevention of NSAID-associated gastric ulcers or duodenal ulcers in patients at risk (age > 60 omeprazole (>65 pantoprazole), previous history of gastric and duodenal ulcers, and previous history of upper GI bleeding.

**Table 5:** Distribution of indicated and non-indicated PPIs among 190 PPI-prescribed study participants’ covariables and associations with non-indicated PPI prescriptions (three patients with uncertain allocation excluded).

Variables	PPI with Indication	PPI without Indication	PPI without Indication Associations	Odds Ratio	95% CI	*p*-Value
Age < 75	23	7	Age	0.98	[0.94–1.03]	0.585
75–90	91	38				
>90	24	7				
Level of care dependency * none	2	0	Level of care dependency * (ref. none, level 0 and level 1)			
0	1	0				
1 considerable	24	12				
2 severe	76	25	level 2	0.76	[0.41–3.75]	0.700
3 most severe	35	15	level 3	0.40	[0.44–4.71]	0.544
Study center Halle (Saale)	49	14	Study center Halle (Saale) (ref. Halle (Saale))			
Lübeck	34	9	Lübeck	0.65	[0.29–4.12]	0.923
Witten/Herdecke	55	29	Witten/Herdecke	1.90	[0.16–1.14]	0.079
Chronic diseases number ≤5	31	11	chronic diseases (ref. ≤ 5)	1.07	[0.98–1.25]	0.338
>5–10	75	33				
>10	32	8				
Antidepressants	39	19	Antidepressants (ref. none)	1.46	[0.98–1.62]	0.112
Antipsychotics	116	42	Antipsychotics (ref. none)	0.81	[0.26–2.12]	0.585
Antidementives	9	3	Antidementives (ref. none)	0.89	[0.17–1.78]	0.076
Antiparkinsonians	13	4	Antiparkinsonians (ref. none)	0.47	[0.12–1.46]	0.172
Cognitive impairment **	73	32	Cognitive impairment ** (ref. none)	1.34	[0.43–2.09]	0.678
Contacts *** family doctor 0	23	15	Contacts *** family doctor (ref. none)	0.93	[0.79–1.11]	0.299
1–2	78	19				
3–4	15	8				
≥5	22	10				
Contacts *** specialists 0	43	20	Contacts *** specialists (ref. none)	1.27	[0.73–1.89]	0.513
1–2	71	24				
3–4	6	1				
No information	18	7				
Number of drugs <5	16	5	Number of drugs (ref. < 5)	1.32	[1.18–1.62]	0.013
5–10	96	37				
>10	26	10				

* Level of care dependency categories: residents’ need for care was assessed by the medical service of the German social care insurance; need for care in performing activities of daily living and household tasks was defined as Level 0: <90 min/day, Level 1: at least 90 min/day, Level 2: at least 3 h/day, and Level 3: at least 4 h/day. ** Residents with cognitive impairment (DSS > 4 (DSS: Dementia Screening Scale (total score ranges from 0 to 14; higher scores indicate more severe cognitive impairments)). *** contacts refer to a physician contact over the last three months.

**Table 6:** Overdoses (>20 mg/d) of up to two to fourfold higher, especially with prophylactic PPI prescriptions according to guideline recommendations, which involve years or decades of PPI use in these constellations.

Indication * (According to Table 4)	PPI	Overdosage (Instead of Prophylactic 20 mg/d **)	Number = 54
diclofenac + ASA ld *** + age ≥ 65 (60) years	pantoprazole	40 mg/d	2
ibuprofen + prednisolone + phenprocoumon + age ≥ 65 (60) years	pantoprazole	40 mg/d	1
ASA ld + age ≥ 65 years	pantoprazole	40 mg/d	28
ASA ld + age ≥ 65 years	omeprazole	40 mg/d	9
ASA ld + age ≥ 65 years	esomeprazole	40 mg/d	2
ASA ld + age ≥ 65 years	pantoprazole	80 mg/d	2
ASA ld + age ≥ 65 years	omeprazole	80 mg/d	1
ASA ld + age ≥ 65 years	esomeprazole	80 mg/d	1
clopidogrel + age ≥ 65 years	pantoprazole	80 mg/d	1
rivaroxaban + age ≥ 65 years	pantoprazole	40 mg/d	2
apixaban + age ≥ 65 years	pantoprazole	40 mg/d	1
clopidogrel ****+ ASA ld	pantoprazole	40 mg/d	3
clopidogrel ***** + ASA ld	omeprazole	80 mg/d	1

* indications according to valid SmPC (Summary of Product Characteristics) and German guideline recommendations at time of investigation and current. ** mg/d = mg per a day. *** ASA ld = 100 mg/day = ASA low-dose. Authors’ note: Cave: ASA ld in combination with NSAIDs no sufficient platelet aggregation inhibition. It is important to take ASA with a time delay before NSAIDs. **** Authors’ note: Cave: in this combination, clopidogrel needs to be taken 12 h apart from pantoprazole. ***** Authors’ note: Cave: clopidogrel should not be combined with omeprazole due to reduced activation of clopidogrel as a prodrug.

## Data Availability

The research data set generated for this study is available on request.
